# Differential Effects of Morning Versus Afternoon Accelerated High-Frequency Repetitive Transcranial Magnetic Stimulation on Sleep Outcomes in Hospitalised Patients With Schizophrenia: Retrospective Cohort Study

**DOI:** 10.62641/aep.v54i3.2227

**Published:** 2026-06-15

**Authors:** Yufeng Xiong, Yan Zhang, Haoshui Hua, Jixin Lin, Mengting Li, Qicheng Zhang, Linlin Jiang

**Affiliations:** ^1^Department of Geriatric Psychiatry, The Third People’s Hospital of Fuyang, 311400 Hangzhou, Zhejiang, China; ^2^Department of Rehabilitation, The Third People’s Hospital of Fuyang, 311400 Hangzhou, Zhejiang, China

**Keywords:** schizophrenia, transcranial magnetic stimulation, circadian rhythm, sleep disorders

## Abstract

**Background:**

This study aimed to investigate the differential effects of accelerated high-frequency repetitive transcranial magnetic stimulation (aHF-rTMS) administered in the morning and afternoon on sleep outcomes in hospitalised patients with schizophrenia.

**Methods:**

This single-centre retrospective cohort study was based on existing inpatient records from the Department of Psychiatry at The Third People’s Hospital of Fuyang between November 2023 and May 2025. Eligible cases were identified from electronic medical records, nursing documentation and aHF-rTMS treatment logs. Patients were divided into a morning aHF-rTMS group and an afternoon aHF-rTMS group according to the predominant treatment-time category documented during hospitalisation. Sleep quality was assessed using the Pittsburgh Sleep Quality Index (PSQI), and nighttime sleep duration was obtained from nursing records. Psychiatric symptoms and cognitive function were assessed using the Positive and Negative Syndrome Scale (PANSS) and Montreal Cognitive Assessment (MoCA), respectively. The influencing factors of sleep outcomes at discharge were explored by multivariate linear regression analysis, and sensitivity analysis was performed using propensity score matching.

**Results:**

The two groups were comparable in baseline demographics, sleep parameters and psychiatric symptoms. During hospitalisation, both groups showed substantial improvement in sleep quality and psychiatric symptoms compared with those upon admission. The morning aHF-rTMS group had significantly lower total PSQI scores at discharge (*p* < 0.001) and longer nighttime sleep duration (β = 0.58, *p* < 0.001) than the afternoon aHF-rTMS group. Sensitivity analysis showed that morning aHF-rTMS remained associated with low total PSQI scores at discharge (β = −1.83, *p* < 0.001). No statistically significant differences in PANSS total scores and MoCA scores at discharge were found between the two groups (both *p* > 0.05). The overall incidence of adverse reactions was 44.44%, slightly higher in the morning group than in the afternoon group (54.02% vs. 35.48%, *p* = 0.012).

**Conclusions:**

The timing of aHF-rTMS treatment may be associated with sleep-related treatment outcomes in hospitalised patients with schizophrenia. Compared with afternoon treatment, morning aHF-rTMS was associated with better subjective sleep quality and longer nighttime sleep duration.

## Introduction

Schizophrenia is a severe mental disorder characterised by perceptual, emotional and behavioural abnormalities. Disorganised thinking and emotional responses are its typical symptoms. Its global prevalence is approximately 0.28%, a low prevalence but a heavy disease burden [1, 2]. In addition to positive and negative symptoms, sleep disorders are considered a highly common but long-underestimated important clinical problem in schizophrenia. Over 50% of patients with schizophrenia experience various forms of sleep problems at different stages of the disease, including prolonged sleep latency, decreased sleep efficiency, reduced slow-wave sleep and circadian rhythm disruption, all of which contribute to reduced sleep quality [3, 4, 5].

Sleep disorders are closely related to symptom severity, cognitive impairment, disease relapse and mortality risk in patients with psychosis [6, 7, 8]. Longitudinal studies indicate that sleep abnormalities may occur before the exacerbation of psychotic symptoms and may participate in the progression and deterioration of the disease by affecting mood regulation, synaptic plasticity and neuroinflammatory processes [9, 10]. Therefore, improving sleep quality and sleep rhythm is a potential important therapeutic target in the comprehensive intervention of schizophrenia.

Antipsychotic drugs have a definite curative effect on controlling positive symptoms; however, their effects on sleep-wake function are heterogeneous, and some drugs are associated with daytime somnolence and circadian rhythm alterations [11, 12]. Against this backdrop, nonpharmacological neuromodulation techniques have gradually attracted attention. As a noninvasive neuromodulation method, repetitive transcranial magnetic stimulation (rTMS) has established efficacy in depressive disorders and shown potential benefits for negative symptoms, auditory hallucinations and cognitive dysfunction in schizophrenia [13, 14, 15].

High-frequency rTMS (≥ 5 Hz) enhances cortical excitability, especially in the dorsolateral prefrontal cortex (DLPFC) region, and has a certain regulating effect on the function of the limbic system in the prefrontal cortex [16]. With its increased number of stimulations per day and shortened treatment cycle, accelerated high-frequency rTMS (aHF-rTMS) has been proposed for the rapid induction of neuroplasticity changes [17, 18]. aHF-rTMS has certain advantages in terms of the time to onset of therapeutic effects and safety in interventions for depressive disorders [19, 20].

Compared with stimulation parameters, the influence of stimulation time (morning or afternoon) on treatment efficacy lacks systematic research. Related neuroscience studies showed significant circadian rhythm characteristics in cortical excitability and neurotransmitter release [21, 22, 23]. Furthermore, the implementation of neuromodulation interventions at different times may produce differences due to variations in endogenous rhythm states, thereby affecting the regulatory role of neural function [24]. Sleep quality problems caused by circadian rhythm disturbances are prominent in patients with schizophrenia, making “treatment time” a potential but not yet fully explored factor in the implementation of neuromodulation interventions [25].

To address this issue with clinical practical significance but insufficient evidence, this study employed a single-centre retrospective cohort design to examine whether the predominant timing of aHF-rTMS during hospitalisation was associated with sleep outcomes in patients with schizophrenia. Multivariate linear regression and propensity score matching (PSM) were used to minimise confounding. The study hypothesised that the predominant morning administration of aHF-rTMS is associated with better sleep-related outcomes than predominant afternoon administration.

## Methods

### Study Design

This study is a single-centre retrospective cohort control research. The study subjects were patients hospitalised in the Department of Psychiatry at The Third People’s Hospital of Fuyang from November 2023 to May 2025. Eligible cases were identified from existing electronic medical records, nursing documentation and rTMS treatment logs according to predefined eligibility criteria. All data were anonymised, and no exposure of patient privacy or additional risk was present. The study protocol was reviewed and approved by the Medical Ethics Committee of The Third People’s Hospital of Fuyang (ethical approval number: (2026)-(2-002-01), and written informed consent was obtained from all participants. The study was conducted in accordance with the Declaration of Helsinki and relevant local ethical requirements [26].

### Participants and Eligibility

#### Inclusion and Exclusion Criteria

Hospitalised patients with a discharge diagnosis of schizophrenia were screened from inpatient records. Diagnostic classification was based on the International Classification of Diseases, Tenth Revision (ICD-10) [27].

Records were eligible if patients: (1) were aged 18–65 years; (2) had received standardised aHF-rTMS for approximately 2–4 weeks during hospitalisation; (3) had available Pittsburgh Sleep Quality Index (PSQI) data at admission and discharge [28]; and (4) had a relatively stable antipsychotic regimen during hospitalisation as documented in the medical record.

Records were excluded if they documented: (1) severe neurological diseases or organic brain diseases; (2) severe physical diseases that may significantly affect sleep assessment; (3) electroconvulsive therapy or other brain stimulation treatment during hospitalisation; or (4) lack of key clinical data or inability to classify the treatment time as morning or afternoon.

After the screening of available records using these predefined criteria, 180 patients were included.

### Grouping

#### Treatment Time Grouping Principles

The exposure group was based on the predominant timing category of aHF-rTMS sessions recorded in the treatment logs during hospitalisation. Patients whose treatment records had ≥ 80% of sessions in the morning window (approximately 8:30–12:30) were classified as the morning aHF-rTMS group, and those with ≥ 80% of sessions in the afternoon window (approximately 14:30–18:30) were classified as the afternoon aHF-rTMS group. In the actual inpatient clinical work, most patients are usually treated twice a day within the same nominal period; however, a small number of patients’ treatment records crossed the morning/afternoon boundary because of ward arrangement, equipment occupation or individual clinical conditions. Therefore, this study used the predominant treatment period rather than absolute attribution of every session for grouping. This method aims to reflect the main circadian rhythm exposure patterns of patients as much as possible under real-world scheduling conditions.

### Accelerated High-Frequency Repetitive Transcranial Magnetic Stimulation (aHF-rTMS) Treatment Procedure and Safety Monitoring

All included cases received adjuvant aHF-rTMS based on conventional antipsychotic treatment. The “8” coil was used for stimulation, and the target was the DLPFC. The stimulation frequency was set at 10 Hz, and the initial stimulation intensity was 110% of the resting motion threshold. If the patient’s tolerance is limited in clinical treatment, then a small tolerance adjustment can be made according to conventional practice.

Each aHF-rTMS treatment contained 40 stimulation sequences, each lasting for 4 s, and the intertrain interval was 26 s. At 10 Hz, each sequence outputted 40 pulses, so 1600 pulses were outputted per treatment. Patients usually receive two treatments twice a day, with an interval of approximately 2 h, and the corresponding theoretical total number of pulses per day is 3200 [16, 18, 29]. Treatment was delivered according to routine clinical practice by trained medical and nursing staff. Tolerability-related information and adverse events were identified from documented clinical records. Except for the treatment-time category, the stimulation target, coil type, frequency, intensity setting, train structure and daily session schedule were consistent between the groups. As a retrospective inpatient cohort, the cumulative total number of sessions and total pulses over the full treatment course could vary across the patients according to clinical length of stay and treatment completion and therefore were not fixed at a single course-level.

### Observation Indicators

#### Sleep-Related Indicators

Pittsburgh Sleep Quality Index (PSQI) scores at admission (T0) and discharge (T1) were extracted from routine medical records. PSQI is a self-report questionnaire comprising 19 items, aggregated into seven component scores: subjective sleep quality, sleep latency, sleep duration, habitual sleep efficiency, sleep disturbances, sleep medication use and daytime dysfunction. Each component is scored from 0 to 3. The total PSQI score is the sum of these seven component scores, ranging from 0 to 21, with a high total score indicating poor overall sleep quality. It has demonstrated good reliability and validity in Chinese populations [28]. Given that this study is retrospective, information can only be extracted from existing records. Only the PSQI total score is always available, and some patients cannot obtain the scores of each part. Nighttime sleep duration documented in nursing records was used as an additional sleep-related indicator.

#### Assessment of Mental Symptoms

Positive and Negative Syndrome Scale (PANSS) scores at admission (T0) and discharge (T1) were retrieved from routine clinical documentation. The PANSS is a 30-item, clinician-rated scale, with items categorised into three subscales: positive symptoms (7 items), negative symptoms (7 items) and general psychopathology (16 items). Each item is scored from 1 (absent) to 7 (extreme). The total score ranges from 30 to 210, and the subscale scores are the sums of their respective items. High scores reflect great symptom severity. PANSS is a widely used clinician-rated instrument in schizophrenia research, and the Chinese Mandarin version has demonstrated good reliability and validity [30]. Cognitive function was screened using the Montreal Cognitive Assessment (MoCA), a 30-point test covering multiple cognitive domains, including attention, memory, language and executive function. A score below 26 suggests possible impairment, and the tool has established reliability and validity for cognitive screening in Chinese patients [31].

#### Adverse Reaction Assessment

Adverse reactions during hospitalisation were ascertained from documented adverse-event entries in the medical records and categorised as headache, dizziness, induced mania/hypomania or other types.

### Statistical Analysis

Statistical analysis was performed using R software (4.5.1, R Foundation for Statistical Computing, Vienna, Austria). Continuous variables were summarised as mean ± standard deviation or median (Q_1_, Q_3_) as appropriate according to their distribution. Normality was assessed using Shapiro–Wilk test, together with inspection of histograms and Q–Q plots when necessary. Intergroup comparisons were performed using independent samples t-test or Mann–Whitney U test. Categorical variables were expressed as number of cases and percentages and examined using χ^2^ test or Fisher’s exact test.

Intragroup comparisons of changes from admission (T0) to discharge (T1) were analysed using paired t-tests or Wilcoxon signed-rank tests. In the principal analysis, the total PSQI score at discharge (T1 PSQI) was employed as the dependent variable, and univariate and multivariate linear regression analyses were conducted to explore the influence of group and baseline factors. In the secondary analyses, nursing-sleep-duration of T1 was employed as the dependent variable, and multiple linear regressions were performed to adjust for potential confounding factors. In the multivariate analyses, strongly correlated variables were included in only one variable based on a VIF value less than 5 to avoid multicollinearity.

In the sensitivity analysis, variables with a standardised mean difference (SMD) greater than 0.1 were further subjected to 1:1 PSM with a 0.2 caliper value to verify the robustness of the results, and the main analysis was repeated in the matched samples. A two-tailed test was performed, and a *p*-value < 0.05 was considered statistically significant.

A post-hoc power analysis for T1 PSQI was conducted based on the observed effect size (Cohen’s d = 0.72) and the actual sample sizes (morning: n = 87; afternoon: n = 93), indicating a statistical power of approximately 0.998 at a two-sided α = 0.05.

## Results

### Baseline Characteristics of Study Participants

A total of 180 eligible inpatient cases were included after record screening, including 87 in the morning aHF-rTMS group and 93 in the afternoon aHF-rTMS group. No statistically significant differences in age, sex, education level, disease duration, body mass index, smoking history and history of hypertension were observed between the two groups (all *p* > 0.05). The two groups were also comparable in terms of sleep quality and psychiatric symptoms at admission. No statistically significant differences in baseline PSQI total score, sleep duration, PANSS total score and subscale scores were found between the two groups. Furthermore, no differences in baseline cognitive function (MoCA) were observed between the two groups (Table 1).

**Table 1.** **Baseline characteristics of patients receiving morning versus afternoon aHF-rT**.

Variables		Total (n = 180)	Afternoon aHF-rTMS (n = 93)	Morning aHF-rTMS (n = 87)	Statistic	*p*	SMD
BMI (kg/m^2^), Mean ± SD		24.06 ± 3.15	24.19 ± 3.08	23.92 ± 3.24	t = 0.57	0.570	–0.08
T0 Sleep Duration (hours), Mean ± SD		6.07 ± 0.97	6.05 ± 0.97	6.09 ± 0.99	t = –0.23	0.818	0.03
T0 Negative Symptoms (points), Mean ± SD		24.07 ± 3.19	24.25 ± 3.08	23.89 ± 3.30	t = 0.76	0.448	–0.05
T0 Positive Symptoms (points), Mean ± SD		20.11 ± 2.75	20.19 ± 2.72	20.01 ± 2.79	t = 0.44	0.658	–0.05
T0 General Psychopathology (points), Mean ± SD		35.36 ± 4.25	35.65 ± 4.20	35.06 ± 4.30	t = 0.93	0.355	–0.14
Age (years), M (Q_1_, Q_3_)		40.50 (28.00, 51.00)	40.00 (28.00, 47.00)	41.00 (30.00, 53.00)	Z = –0.49	0.621	0.08
Disease Duration (years), M (Q_1_, Q_3_)		5.85 (3.80, 9.80)	5.80 (3.90, 9.80)	5.90 (3.40, 9.00)	Z = –0.32	0.751	–0.04
Chlorpromazine Equivalent (mg/day), M (Q_1_, Q_3_)		300.00 (200.00, 425.00)	300.00 (200.00, 400.00)	400.00 (200.00, 500.00)	Z = –1.11	0.265	0.14
NLR, M (Q_1_, Q_3_)		1.81 (1.45, 2.39)	1.81 (1.44, 2.36)	1.81 (1.45, 2.50)	Z = –0.33	0.741	0.09
TSH (mIU/L), M (Q_1_, Q_3_)		2.23 (1.34, 3.03)	2.18 (1.24, 3.24)	2.23 (1.39, 2.87)	Z = –0.62	0.536	–0.12
T0 PSQI Total (points), M (Q_1_, Q_3_)		11.00 (10.00, 13.00)	11.00 (10.00, 13.00)	11.00 (10.00, 12.00)	Z = –0.46	0.640	–0.05
T0 PANSS Total (points), M (Q_1_, Q_3_)		80.00 (74.00, 85.00)	81.00 (75.00, 85.00)	78.00 (73.50, 84.00)	Z = –1.05	0.292	–0.15
T0 MoCA (points), M (Q_1_, Q_3_)		24.00 (21.75, 26.00)	24.00 (22.00, 26.00)	23.00 (21.00, 25.50)	Z = –1.18	0.237	–0.13
Gender, n (%)					χ^2^ = 0.67	0.413	
	Male	109 (60.56)	59 (63.44)	50 (57.47)			–0.12
	Female	71 (39.44)	34 (36.56)	37 (42.53)			0.12
Smoking, n (%)					χ^2^ = 1.09	0.297	
	No	113 (62.78)	55 (59.14)	58 (66.67)			0.16
	Yes	67 (37.22)	38 (40.86)	29 (33.33)			–0.16
Education Level, n (%)					χ^2^ = 4.22	0.121	
	Junior high or below	43 (23.89)	28 (30.11)	15 (17.24)			–0.34
	Senior high	101 (56.11)	47 (50.54)	54 (62.07)			0.24
	College or above	36 (20.00)	18 (19.35)	18 (20.69)			0.03
Hypertension, n (%)					χ^2^ = 0.03	0.861	
	No	156 (86.67)	81 (87.10)	75 (86.21)			–0.03
	Yes	24 (13.33)	12 (12.90)	12 (13.79)			0.03
Sedation Effect, n (%)					χ^2^ = 0.15	0.702	
	No	76 (42.22)	38 (40.86)	38 (43.68)			0.06
	Yes	104 (57.78)	55 (59.14)	49 (56.32)			-0.06
Baseline Hypnotic, n (%)					χ^2^ = 0.07	0.793	
	No	134 (74.44)	70 (75.27)	64 (73.56)			–0.04
	Yes	46 (25.56)	23 (24.73)	23 (26.44)			0.04

Note: Data are presented as mean ± SD, median (Q_1_, Q_3_), or n (%), as appropriate. Continuous variables were compared using the independent-samples t test or Mann–Whitney U test, and categorical variables were compared using the chi-square test. BMI is expressed in kg/m^2^; sleep duration in hours; chlorpromazine equivalent in mg/day; TSH in mIU/L; PSQI, PANSS, and MoCA scores in points. Abbreviations: aHF-rTMS, accelerated high-frequency repetitive transcranial magnetic stimulation; BMI, body mass index; T0, at admission; PSQI, Pittsburgh Sleep Quality Index; PANSS, Positive and Negative Syndrome Scale; MoCA, Montreal Cognitive Assessment; NLR, neutrophil-to-lymphocyte ratio; TSH, thyroid-stimulating hormone; SD, standard deviation; SMD, standardized mean difference; Q_1_, first quartile; Q_3_, third quartile.

### Intra- and Intergroup Comparisons from Admission to Discharge

#### Changes in Sleep-Related Indicators

During hospitalisation, sleep patterns improved in both groups compared with those upon admission. In the afternoon aHF-rTMS group, the recorded sleep duration at discharge was significantly longer than at admission, and the total PSQI score decreased significantly (both *p* < 0.001). Similar changes were observed in the morning aHF-rTMS group, but the increase in sleep duration and the decrease in total PSQI score were relatively greater.

Intergroup comparisons showed that the morning aHF-rTMS group had a lower total PSQI score at discharge than the afternoon aHF-rTMS group, indicating a more favourable subjective sleep outcome associated with morning treatment timing (Table 2).

**Table 2.** **Changes in sleep and psychiatric symptoms from admission to discharge**.

Variables	Group	T0	T1	Wilcoxon signed ranks test/paired t test	*p*
Sleep_Duration (hours)	Afternoon aHF-rTMS	6.05 ± 0.97	6.61 ± 1.02	–9.72	<0.001
	Morning aHF-rTMS	6.09 ± 0.99	7.21 ± 1.19	–19.76	<0.001
	t	–0.23	–3.61		
	*p*	0.818	<0.001		
PSQI_Total (points)	Afternoon aHF-rTMS	11.00 (10.00, 13.00)	8.61 ± 2.72	–7.88	<0.001
	Morning aHF-rTMS	11.00 (10.00, 12.00)	6.70 ± 2.59	–8.12	<0.001
	t/z	–0.46	4.82		
	*p*	0.640	<0.001		
PANSS_Total (points)	Afternoon aHF-rTMS	81.00 (75.00, 85.00)	64.00 (60.00, 69.00)	–8.39	<0.001
	Morning aHF-rTMS	78.00 (73.50, 84.00)	63.00 (58.00, 68.00)	–8.11	<0.001
	z	–1.05	–1.07		
	*p*	0.292	0.285		
Negative_Symptoms (points)	Afternoon aHF-rTMS	24.25 ± 3.08	19.37 ± 2.66	55.71	<0.001
	Morning aHF-rTMS	23.89 ± 3.30	19.06 ± 2.66	43.92	<0.001
	t	0.76	0.78		
	*p*	0.448	0.439		
Positive_Symptoms (points)	Afternoon aHF-rTMS	20.19 ± 2.72	16.00 (14.00, 18.00)	–8.53	<0.001
	Morning aHF-rTMS	20.01 ± 2.79	16.00 (14.50, 17.00)	–8.24	<0.001
	t/z	0.44	–0.68		
	*p*	0.658	0.497		
General Psychopathology (points)	Afternoon aHF-rTMS	35.65 ± 4.20	28.26 ± 3.57	53.02	<0.001
	Morning aHF-rTMS	35.06 ± 4.30	27.95 ± 3.70	47.12	<0.001
	t	0.93	0.56		
	*p*	0.355	0.576		
MoCA_Score (points)	Afternoon aHF-rTMS	24.00 (22.00, 26.00)	25.00 (23.00, 27.00)	–6.48	<0.001
	Morning aHF-rTMS	23.00 (21.00, 25.50)	24.00 (21.50, 27.00)	–5.46	<0.001
	z	–1.18	–1.19		
	*p*	0.237	0.232		

Note: Data are presented as mean ± SD or median (Q_1_, Q_3_), as appropriate. T0 indicates admission and T1 indicates discharge. Within-group comparisons were performed using the paired t test or Wilcoxon signed-rank test, as appropriate; between-group comparisons at each time point were performed using the independent-samples t test or Mann–Whitney U test. Sleep_Duration is expressed in hours; PSQI_Total, PANSS_Total, Negative_Symptoms, Positive_Symptoms, General Psychopathology, and MoCA_Score are expressed in points. Abbreviations: aHF-rTMS, accelerated high-frequency repetitive transcranial magnetic stimulation; PSQI, Pittsburgh Sleep Quality Index; PANSS, Positive and Negative Syndrome Scale; MoCA, Montreal Cognitive Assessment; SD, standard deviation; Q_1_, first quartile; Q_3_, third quartile.

#### Changes in Mental Symptoms

During hospitalisation, the total PANSS score and the scores of the positive, negative and general psychopathology subscales in both groups decreased significantly compared with those upon admission (all P < 0.001), indicating an overall improvement in mental symptoms. However, in the intergroup comparisons of various PANSS indicators at discharge, the differences between the two groups did not reach statistical significance (both *p* > 0.05, Table 2).

#### Changes in Cognitive Function

Although the MoCA scores of both groups at discharge were higher than those at admission (*p* < 0.001), no statistically significant difference in MoCA scores was found between the two groups (*p* > 0.05). This finding indicates that the observed association between treatment timing and sleep outcomes during this hospitalisation window was not accompanied with significant intergroup differences in cognitive screening indicators (Table 2).

### Analysis of Factors Influencing Sleep Quality at Discharge

Univariate linear regression analysis with the total PSQI score at discharge (T1 PSQI) as the dependent variable showed that the duration of aHF-rTMS treatment was associated with sleep quality at discharge. Compared with afternoon aHF-rTMS, morning aHF-rTMS was associated with a lower total PSQI score at discharge. Furthermore, gender, education, baseline hypnotherapy use, sleep duration and total PSQI score at admission were also associated with PSQI at discharge.

In a multivariate linear regression analysis incorporating potential confounding factors, T0 sleep duration and T0 MoCA were removed to avoid multicollinearity, as these were collinear with T0 PSQI/PANSS. After the adjustment for potential confounders, including sex, education level, baseline hypnotherapy use and total PSQI score at admission, morning aHF-rTMS, baseline PSQI and sex were identified as independent influencing factors of T1 PSQI (Table 3). Specifically, morning aHF-rTMS remained significantly associated with a lower T1 PSQI score compared with afternoon aHF-rTMS (β = −1.84, *p* < 0.001) (Table 3).

**Table 3.** **Univariate and multivariate linear regression analyses of PSQI score at discharge**.

Variables		Univariate				Multivariate			
		SE	t	*p*	β (95%CI)	SE	t	*p*	β (95%CI)
Group									
	Afternoon aHF-rTMS				0.00 (Reference)				0.00 (Reference)
	Morning aHF-rTMS	0.40	–4.82	<0.001	–1.91 (–2.69– –1.13)	0.25	–7.23	<0.001	–1.84 (–2.34––1.34)
Gender									
	Male				0.00 (Reference)				0.00 (Reference)
	Female	0.43	2.13	0.034	0.91 (0.07–1.74)	0.26	3.19	0.002	0.83 (0.32–1.34)
Smoking									
	No				0.00 (Reference)				
	Yes	0.44	–0.12	0.907	–0.05 (–0.91–0.80)				
Education Level									
	Junior high or below				0.00 (Reference)				0.00 (Reference)
	Senior high	0.51	–1.21	0.226	–0.62 (–1.61–0.38)	0.31	–0.77	0.441	–0.24 (–0.85–0.37)
	College or above	0.63	–2.49	0.014	–1.57 (–2.81– –0.34)	0.39	–1.49	0.138	–0.58 (–1.33–0.18)
Hypertension									
	No				0.00 (Reference)				
	Yes	0.62	0.81	0.417	0.50 (–0.71–1.72)				
Sedation Effect									
	No				0.00 (Reference)				
	Yes	0.43	–0.78	0.435	–0.33 (–1.17–0.50)				
Baseline Hypnotic									
	No				0.00 (Reference)				0.00 (Reference)
	Yes	0.47	2.67	0.008	1.26 (0.34–2.19)	0.29	2.65	0.009	0.77 (0.20–1.35)
Disease Duration		0.05	–0.11	0.910	–0.01 (–0.11–0.10)				
Age		0.02	0.24	0.807	0.00 (–0.03–0.04)				
BMI		0.07	–0.22	0.829	–0.01 (–0.15–0.12)				
Chlorpromazine Equivalent		0.01	–0.34	0.737	–0.01 (–0.02–0.02)				
NLR		0.26	–0.36	0.723	–0.09 (–0.60–0.42)				
TSH		0.17	0.67	0.504	0.11 (–0.21–0.44)				
T0 Sleep Duration		0.19	–7.91	<0.001	–1.48 (–1.84– –1.11)				
T0 PSQI Total		0.07	13.58	<0.001	0.94 (0.80–1.08)	0.06	14.96	<0.001	0.90 (0.78–1.01)
T0 PANSS Total		0.03	0.42	0.676	0.01 (–0.04–0.07)				
T0 Negative Symptoms		0.07	0.24	0.810	0.02 (–0.11–0.15)				
T0 Positive Symptoms		0.08	0.73	0.466	0.06 (–0.09–0.21)				
T0 General Psychopathology		0.05	0.08	0.939	0.00 (–0.09–0.10)				
T0 MoCA Score		0.07	0.28	0.782	0.02 (–0.12–0.16)				

Note: Univariate and multivariate linear regression analyses were performed with PSQI score at discharge as the dependent variable. β indicates the unstandardized regression coefficient, and P values are two-sided. BMI is expressed in kg/m^2^; chlorpromazine equivalent in mg/day; T0 sleep duration in hours; TSH in mIU/L; PSQI, PANSS, and MoCA scores in points. Abbreviations: SE, standard error; CI, confidence interval; aHF-rTMS, accelerated high-frequency repetitive transcranial magnetic stimulation; BMI, body mass index; NLR, neutrophil-to-lymphocyte ratio; TSH, thyroid-stimulating hormone; PSQI, Pittsburgh Sleep Quality Index; PANSS, Positive and Negative Syndrome Scale; MoCA, Montreal Cognitive Assessment; T0, at admission.

### Hierarchical Analysis of Sleep Duration at Discharge

A hierarchical linear regression analysis was conducted using sleep duration recorded in nursing records at discharge as the dependent variable. In the unadjusted model and the model with stepwise adjustments for demographic, clinical and pharmacological factors, morning aHF-rTMS was associated with long sleep duration at discharge. This association was consistent across different models, suggesting a relatively stable relationship between morning aHF-rTMS administration and prolonged sleep duration (β = 0.58, *p* < 0.001) (Table 4).

**Table 4.** **Significance test of stepwise regression model**.

Variables		Model 1		Model 2		Model 3	
		β (95%CI)	*p*	β (95%CI)	*p*	β (95%CI)	*p*
Group							
	Afternoon aHF-rTMS	0.00 (Reference)		0.00 (Reference)		0.00 (Reference)	
	Morning aHF-rTMS	0.59 (0.27–0.91)	<0.001	0.60 (0.28–0.93)	<0.001	0.58 (0.42–0.75)	<0.001

Note: The dependent variable was sleep duration at discharge, expressed in hours. β indicates the unstandardized regression coefficient, and P values are two-sided. Model 1 was unadjusted. Model 2 was adjusted for gender, age, and BMI. Model 3 was further adjusted for gender, smoking, education level, hypertension, sedation effect, baseline hypnotic use, age, disease duration, BMI, chlorpromazine equivalent, NLR, TSH, T0 sleep duration, and T0 PANSS total score. Age and disease duration are expressed in years; BMI in kg/m^2^; chlorpromazine equivalent in mg/day; TSH in mIU/L; PANSS score in points. Abbreviations: CI, confidence interval; aHF-rTMS, accelerated high-frequency repetitive transcranial magnetic stimulation; BMI, body mass index; NLR, neutrophil-to-lymphocyte ratio; TSH, thyroid-stimulating hormone; PANSS, Positive and Negative Syndrome Scale; T0, at admission.

### Adverse Reactions

Documented adverse reactions occurred in 44.44% of included cases. The incidence of adverse reactions was higher in the morning aHF-rTMS group than in the afternoon aHF-rTMS group (54.02% vs. 35.48%, *p* = 0.012). Headache and dizziness were the most common types of adverse reactions. The incidence of mania or hypomania was low, and no serious adverse events were observed (Table 5).

**Table 5.** **Adverse reactions associated with aHF-rTMS in the two groups**.

Variables		Total (n = 180)	Afternoon aHF-rTMS (n = 93)	Morning aHF-rTMS (n = 87)	Statistic	*p*
Adverse Reaction, n(%)					χ^2^ = 6.26	0.012
	No	100 (55.56)	60 (64.52)	40 (45.98)		
	Yes	80 (44.44)	33 (35.48)	47 (54.02)		
Adverse Reaction cat, n(%)					-	0.736
	None	100 (55.56)	50 (53.76)	50 (57.47)		
	Headache	45 (25.00)	26 (27.96)	19 (21.84)		
	Dizziness	31 (17.22)	16 (17.20)	15 (17.24)		
	Mania/hypomania	1 (0.56)	0 (0.00)	1 (1.15)		
	Other	3 (1.67)	1 (1.08)	2 (2.30)		

Note: Data are presented as n (%). Categorical variables were compared using the chi-square test or Fisher’s exact test, as appropriate. “Adverse Reaction cat” refers to the classification of documented adverse reactions during treatment. Abbreviations: aHF-rTMS, accelerated high-frequency repetitive transcranial magnetic stimulation; χ^2^, chi-square test.

### Sensitivity Analysis

**Fig. 1.**
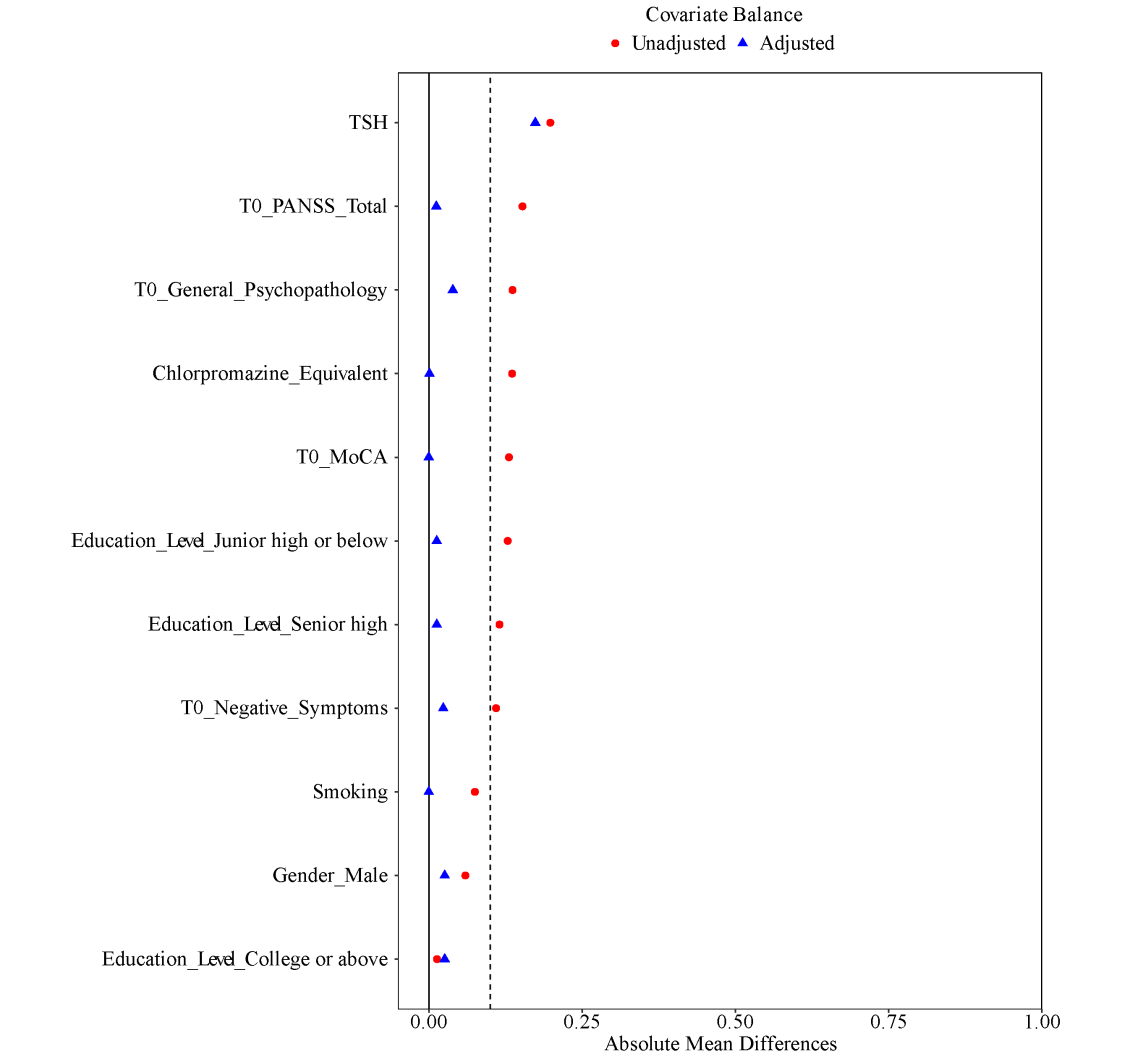
**Standardized mean differences of baseline covariates before and after propensity score matching**. Fig. 1 illustrates the standardized mean differences (SMDs) of baseline covariates before and after propensity score matching. Each point represents the SMD of an individual covariate. Values closer to zero indicate better balance between the morning and afternoon aHF-rTMS groups. MoCA, Montreal Cognitive Assessment; PANSS, Positive and Negative Syndrome Scale.

PSM was performed on variables with baseline characteristics having an SMD greater than 0.1. After matching, the SMD in baseline characteristics between the two groups of patients significantly decreased. (**Supplementary Table 1**, Fig. 1).

After multivariate linear regression analysis was repeated using the matched samples, morning aHF-rTMS remained significantly associated with a decrease in total PSQI score at discharge, and the direction and magnitude of the effect were basically consistent with the main analysis results (β = −1.83, *p* < 0.001) (Table 6).

**Table 6.** **Hierarchical linear regression analysis of PSQI at discharge after propensity score matching**.

Variables		Model 1		Model 2		Model 3	
		β (95%CI)	*p*	β (95%CI)	*p*	β (95%CI)	*p*
Group							
	Afternoon aHF-rTMS	0.00 (Reference)		0.00 (Reference)		0.00 (Reference)	
	Morning aHF-rTMS	–1.88 (–2.73– –1.04)	<0.001	–1.91 (–2.75– –1.06)	<0.001	–1.83 (–2.37– –1.27)	<0.001

Note: The dependent variable was PSQI score at discharge after propensity score matching, expressed in points. β indicates the unstandardized regression coefficient, and P values are two-sided. Model 1 was unadjusted. Model 2 was adjusted for gender, age, and BMI. Model 3 was further adjusted for gender, smoking, education level, hypertension, sedation effect, baseline hypnotic use, age, disease duration, BMI, chlorpromazine equivalent, NLR, TSH, T0 PSQI total score, and T0 PANSS total score. Age and disease duration are expressed in years; BMI in kg/m²; chlorpromazine equivalent in mg/day; TSH in mIU/L; PSQI and PANSS scores in points. Abbreviations: CI, confidence interval; aHF-rTMS, accelerated high-frequency repetitive transcranial magnetic stimulation; BMI, body mass index; NLR, neutrophil-to-lymphocyte ratio; TSH, thyroid-stimulating hormone; PSQI, Pittsburgh Sleep Quality Index; PANSS, Positive and Negative Syndrome Scale; T0, at admission.

## Discussion

Using retrospective inpatient data, this study compared the associations of morning versus afternoon aHF-rTMS timing with sleep outcomes in hospitalised patients with schizophrenia. Results showed that patients receiving morning aHF-rTMS had lower PSQI scores at discharge than those receiving afternoon aHF-rTMS, indicating a relative advantage of morning stimulation timing with respect to subjective sleep outcomes. However, the morning group also showed a higher incidence of documented adverse reactions.

### Possible Mechanisms of Treatment Time and Sleep Improvement

This study identified an association between different treatment time segments and sleep quality outcomes. Previous studies showed that irregular lighting and circadian rhythm signal disorders in psychiatric inpatient environments often lead to sleep disorders, and regular circadian rhythm lighting and structured daily routines help improve clinical symptoms and sleep quality [32]. Basic neuroscience studies suggested that cortical excitability and synaptic plasticity change dynamically in the circadian rhythm cycle; early morning is a sensitive period of transition from sleep to wakefulness, when the brain’s ability to adjust plasticity may be high [22, 33]. At this stage, the application of high-frequency stimulation may bring a lasting regulatory effect beyond the instantaneous effect of stimulation, which is conducive to the formation of the steady-state of the prefrontal cortex-related network, thereby supporting stable cognitive and sleep processes [34]. Additionally, patients with schizophrenia generally have circadian rhythm disorders, including abnormal melatonin secretion, delayed sleep-wake rhythm and decreased circadian amplitude [35, 36]. This phenomenon is closely related to the rhythm disorder of the central circadian pacemaker-suprachiasmatic nucleus (SCN). Changes in circadian rhythm gene expression were also observed in rTMS stimulation target areas such as the prefrontal cortex [25, 37]. As the coordination centre of internal time signals, SCN regulates sleep–wake rhythm through downstream neural and endocrine pathways and synchronises the external light–dark cycle with the body’s physiological behaviour. Morning stimulation may be in line with the sensitive period of these intrinsic rhythm signals, potentially enhancing the synchronisation between the suprachiasmatic nucleus and the cortical network and thereby improving nighttime sleep. This circadian-based hypothesis is consistent with studies on the interaction between neural regulation and rhythmic systems, suggesting that temporal neural stimulation may affect regulatory networks that overlap with intrinsic rhythmic regulation [38]. However, the present study did not directly assess circadian phase, melatonin rhythm, actigraphy or polysomnographic parameters. Therefore, any mechanistic explanation should be considered speculative.

### Comparison with Previous aHF-rTMS Clinical Studies

Studies on rTMS in schizophrenia mainly focused on outcomes such as negative symptoms, auditory hallucinations or mood symptoms and then explored optimal setting parameters [39]. However, research that systematically evaluates sleep outcomes is relatively scarce. “Sleep improvement” is often mentioned as a concomitant benefit rather than a primary endpoint in study design, making it difficult to directly extract referable sleep protocols from literature on rTMS for schizophrenia. Existing evidence from previous works suggests that high-frequency rTMS may improve subjective sleep quality, such as PSQI scores; however, these studies were not conducted on patients with schizophrenia [40, 41, 42], which affects our reference value, as the pathogenic mechanisms are not entirely equivalent to those in the schizophrenia population.

Research evidence for aHF-rTMS mainly comes from the field of depressive disorders. Accelerated protocols typically improve treatment efficiency through “multiple stimulations per day and shortened total treatment duration”, but the efficacy is highly sensitive to factors such as the number of daily treatments, treatment intervals, total pulse dose and target localisation accuracy. In natural cohorts, the twice-daily regimen of 10 Hz DLPFC is similar in remission rate to the traditional once-daily regimen but may result in faster treatment turnover, suggesting that accelerated protocols have practical feasibility and potential cost advantages in hospital settings [43].

The results of this study suggest the importance of treatment timing, which has been almost unmentioned in previous literature on rTMS in schizophrenia but is associated with circadian rhythms. The findings of this study show that it may have value.

### Mental Symptoms and Cognitive Outcomes

Although morning aHF-rTMS showed an advantage in sleep outcomes, no significant differences in PANSS scores and MoCA scores were observed between the two groups. This result was not unexpected. Firstly, the study sample consisted of hospitalised patients with relatively stable baseline mental symptoms and received standardised drug treatment during hospitalisation, which may have weakened the impact of treatment time differences on mental symptoms and cognitive function. Secondly, treatment time is only one factor; we cannot infer that different treatment times significantly affect overall mental and cognitive status and cause differences between the two groups. This situation was not the primary outcome of our study.

Both groups showed changes in their MoCA scores from admission to discharge; this finding requires careful clinical interpretation. These slight improvements are likely attributable to nonspecific factors, such as improved patient attention, cooperation and test participation, and improvements accompanying overall symptom relief. Standard aHF-rTMS was also used. Therefore, the observed changes in MoCA scores are likely to reflect an overall trend of improvement in clinical status rather than a specific effect of aHF-rTMS or treatment duration on cognitive function.

### Clinical Significance and Practical Implications

From a clinical practice perspective, the results of this study suggest that, when resources permit, administering aHF-rTMS in the morning may help optimise sleep-related outcomes. This finding has good operability and is especially applicable to patient groups with relatively adjustable treatment times in hospital settings. Safety should also be noted. Although aHF-rTMS in the morning was more effective in improving sleep quality, the proportion of mild adverse reactions was slightly higher, such as temporary headache or scalp discomfort. No serious adverse events were reported. This result suggests that efficacy and tolerance should be taken into account when formulating treatment plans.

### Study Limitations

This study still has several limitations. Firstly, the retrospective design limits the strength of causal inference. Although PSM and multilevel regression were applied, residual confounding cannot be completely excluded. Treatment timing may have been influenced by ward logistics and patient-specific factors, such as staffing schedules, bed availability, rehabilitation programs, light exposure and group therapy timing. Additionally, unmeasured circadian-related factors, including chronotype, daytime activity level and natural light exposure, were not systematically recorded in the available clinical documentation and may also have influenced treatment-time assignment and sleep outcomes. Secondly, PSQI reflects sleep quality over the previous month, whereas hospitalisation and aHF-rTMS treatment lasted approximately 2–4 weeks in the present study. Therefore, the T1 PSQI obtained at discharge may partially reflect sleep during the early treatment period or even some pre-intervention days, which may have affected the precision of outcome assessment. Additionally, the item-level data required to calculate PSQI component scores were incomplete across the full cohort, limiting the interpretation of specific sleep domains. Thirdly, the sleep outcomes in this study were mainly based on a subjective questionnaire and nursing-recorded sleep duration; objective measures such as polysomnography or actigraphy were lacking. Although these indicators are practical in routine inpatient care, they are less informative for sleep architecture and mechanistic interpretation. Additionally, exposure classification was based on the predominant treatment period rather than the absolute timing of every treatment session, although most patients were treated within the same nominal time window. Fourthly, some potentially relevant proxy variables were not fully included in the analysis. For example, length of stay was not entered as a covariate because it may function as a mediator, rather than a simple confounder, in the relationship between treatment timing and sleep outcomes; adjusting for this variable could underestimate or distort the overall association. Ward conditions, including lighting and layout, were largely uniform, and treatment schedules were generally consistent across weekdays and weekends because on-duty clinical staff were available every day. Finally, this study was based on data from psychiatric inpatients in a single centre. In this setting, treatment arrangements, ward routines, staffing patterns and environmental conditions were relatively structured and standardised. Therefore, the associations observed in this study may not be directly generalisable to outpatient populations, community settings or healthcare systems with different treatment workflows and environmental contexts. Future multicentre prospective studies, including outpatient samples and direct assessments of circadian phenotype and objective sleep measures such as polysomnography, are needed to further evaluate the generalisability of treatment-timing effects and their potential mechanisms.

## Conclusions

This study provides relevant evidence for the long-neglected question of when to administer aHF-rTMS to hospitalised patients with schizophrenia. Compared with afternoon treatment, morning aHF-rTMS was associated with better subjective sleep quality and longer nighttime sleep duration but higher proportion of adverse events. These findings suggest that treatment timing may be a clinically relevant factor when balancing the potential sleep-related benefits and tolerability in hospitalised patients with schizophrenia. Further validation through large-scale, prospective randomised controlled trials is still needed.

## Availability of Data and Materials

The data that support the findings of this study are available from the corresponding author upon reasonable request.

## Supplementary Material

Supplementary material associated with this article can be found, in the online version, at https://doi.org/10.62641/aep.v54i3.2227.
