# Supplementary material for: Differential Effects of Morning Versus Afternoon Accelerated High-Frequency Repetitive Transcranial Magnetic Stimulation on Sleep Outcomes in Hospitalised Patients With Schizophrenia: Retrospective Cohort Study
Source: Actas Esp Psiquiatr. 2026 Jun 15;54(3):612–25. doi: 10.62641/aep.v54i3.2227 (PMC13294763; doi:10.62641/aep.v54i3.2227)
Supplement: Supplementary file 1 [file ActEsp-54-3-612-625-s1.zip › Supplementary Table 1.docx]

**Supplementary Table 1. Baseline characteristics of hospitalized patients with schizophrenia receiving morning versus afternoon aHF-rTMS after propensity score matching.**

| Variable | Total (n = 154) | Afternoon aHF-rTMS (n = 77) | Morning aHF-rTMS (n = 77) | Statistic | *P* | SMD |
| --- | --- | --- | --- | --- | --- | --- |
| BMI, Mean ± SD | 24.15 ± 3.21 | 24.14 ± 3.19 | 24.17 ± 3.25 | t=-0.07 | 0.946 | 0.01 |
| T0 Sleep Duration, Mean ± SD | 6.09 ± 0.97 | 6.07 ± 0.95 | 6.10 ± 0.99 | t=-0.22 | 0.830 | 0.03 |
| T0 PSQI Total, Mean ± SD | 11.00 (10.00, 13.00) | 11.00 (9.00, 13.00) | 11.00 (10.00, 12.00) | Z=-0.39 | 0.697 | -0.05 |
| T0 Negative Symptoms, Mean ± SD | 23.94 ± 3.16 | 23.97 ± 2.94 | 23.90 ± 3.38 | t=0.15 | 0.879 | -0.02 |
| T0 Positive Symptoms, Mean ± SD | 20.05 ± 2.75 | 19.97 ± 2.66 | 20.13 ± 2.85 | t=-0.35 | 0.726 | 0.06 |
| T0 General Psychopathology, Mean ± SD | 35.24 ± 4.41 | 35.32 ± 4.37 | 35.16 ± 4.48 | t=0.24 | 0.813 | -0.04 |
| Age, M (Q₁, Q₃) | 40.50 (28.00, 50.75) | 39.00 (28.00, 46.00) | 43.00 (29.00, 54.00) | Z=-0.71 | 0.475 | 0.13 |
| Disease Duration, M (Q₁, Q₃) | 5.90 (3.80, 9.80) | 5.80 (3.90, 9.60) | 6.20 (3.50, 10.40) | Z=-0.18 | 0.859 | 0.04 |
| Chlorpromazine Equivalent, M (Q₁, Q₃) | 300.00 (200.00, 500.00) | 300.00 (200.00, 500.00) | 400.00 (200.00, 500.00) | Z=-0.08 | 0.937 | 0.00 |
| NLR, M (Q₁, Q₃) | 1.80 (1.44, 2.35) | 1.79 (1.44, 2.27) | 1.81 (1.45, 2.35) | Z=-0.33 | 0.745 | 0.08 |
| TSH, M (Q₁, Q₃) | 2.23 (1.31, 3.02) | 2.18 (1.24, 3.14) | 2.25 (1.32, 2.87) | Z=-0.59 | 0.558 | -0.17 |
| T0 PANSS Total, M (Q₁, Q₃) | 80.00 (73.25, 84.00) | 81.00 (74.00, 84.00) | 78.00 (73.00, 85.00) | Z=-0.04 | 0.967 | -0.01 |
| T0 MoCA, M (Q₁, Q₃) | 24.00 (21.00, 26.00) | 24.00 (21.00, 26.00) | 23.00 (21.00, 26.00) | Z=-0.16 | 0.874 | 0.00 |
| Gender, n (%) |  |  |  | χ²=0.11 | 0.741 |  |
| Male | 94 (61.04) | 48 (62.34) | 46 (59.74) |  |  | -0.05 |
| Female | 60 (38.96) | 29 (37.66) | 31 (40.26) |  |  | 0.05 |
| Smoking, n (%) |  |  |  | χ²=0.00 | 1.000 |  |
| No | 100 (64.94) | 50 (64.94) | 50 (64.94) |  |  | 0.00 |
| Yes | 54 (35.06) | 27 (35.06) | 27 (35.06) |  |  | 0.00 |
| Education Level, n (%) |  |  |  | χ²=0.16 | 0.922 |  |
| Junior high or below | 29 (18.83) | 14 (18.18) | 15 (19.48) |  |  | 0.03 |
| Senior high | 91 (59.09) | 45 (58.44) | 46 (59.74) |  |  | 0.03 |
| College or above | 34 (22.08) | 18 (23.38) | 16 (20.78) |  |  | -0.06 |
| Hypertension, n (%) |  |  |  | χ²=0.21 | 0.645 |  |
| No | 132 (85.71) | 67 (87.01) | 65 (84.42) |  |  | -0.07 |
| Yes | 22 (14.29) | 10 (12.99) | 12 (15.58) |  |  | 0.07 |
| Sedation Effect, n (%) |  |  |  | χ²=0.96 | 0.327 |  |
| No | 64 (41.56) | 29 (37.66) | 35 (45.45) |  |  | 0.15 |
| Yes | 90 (58.44) | 48 (62.34) | 42 (54.55) |  |  | -0.16 |
| Baseline Hypnotic, n (%) |  |  |  | χ²=0.03 | 0.857 |  |
| No | 111 (72.08) | 56 (72.73) | 55 (71.43) |  |  | -0.03 |
| Yes | 43 (27.92) | 21 (27.27) | 22 (28.57) |  |  | 0.03 |

Note: Data are presented as mean ± SD, median (Q_1_, Q_3_), or n (%), as appropriate. Continuous variables were compared using the independent-samples t test or Mann–Whitney U test, and categorical variables were compared using the chi-square test. BMI is expressed in kg/m²; sleep duration in hours; chlorpromazine equivalent in mg/day; TSH in mIU/L; PSQI, PANSS, and MoCA scores in points. Abbreviations: aHF-rTMS, accelerated high-frequency repetitive transcranial magnetic stimulation; BMI, body mass index; T0, at admission; PSQI, Pittsburgh Sleep Quality Index; PANSS, Positive and Negative Syndrome Scale; MoCA, Montreal Cognitive Assessment; NLR, neutrophil-to-lymphocyte ratio; TSH, thyroid-stimulating hormone; SD, standard deviation; SMD, standardized mean difference; Q_1_, first quartile; Q_3_, third quartile.
